# Metagenomic Analysis Revealed Significant Changes in the Beef Cattle Rectum Microbiome Under Fescue Toxicosis

**DOI:** 10.3390/biology14091197

**Published:** 2025-09-05

**Authors:** Gastón F. Alfaro, Yihang Zhou, Wenqi Cao, Yue Zhang, Soren P. Rodning, Russell B. Muntifering, Wilmer J. Pacheco, Sonia J. Moisá, Xu Wang

**Affiliations:** 1 Department of Animal Sciences, College of Agriculture, Auburn University, Auburn, AL 36849, USA; gastonfedericoalfaro@gmail.com (G.F.A.);; 2 Department of Animal Sciences, Faculty of Agricultural Sciences, National University of Córdoba, Córdoba 5000, Argentina; 3 Department of Pathobiology, College of Veterinary Medicine, Auburn University, Auburn, AL 36849, USA; 4 Department of Poultry Science, College of Agriculture, Auburn University, Auburn, AL 36849, USA; wjp0010@auburn.edu; 5 Department of Animal Science, University of Tennessee, Knoxville, TN 37996, USA; 6 Center for Advanced Science, Innovation and Commerce, Alabama Agricultural Experiment Station, Auburn, AL 36849, USA; 7 HudsonAlpha Institute for Biotechnology, Huntsville, AL 35806, USA

**Keywords:** tall fescue, endophyte, gut microbiome, microbial diversity, Ruminococcaceae, antimicrobial resistance

## Abstract

Tall fescue toxicosis, caused by ingestion of endophyte-infected tall fescue (*Lolium arundinaceum*), impairs growth and reproduction in beef cattle and results in over USD 3 billion annual loss to the U.S. livestock industry. While the effects on host metabolism and rumen function have been described, the impact on the rectal microbiome remains poorly understood. In this study, we performed whole-genome shotgun metagenomic sequencing on fecal samples collected before and after a 30-day toxic fescue seed supplementation from eight pregnant Angus × Simmental cows and heifers. We generated 157 Gbp of sequencing data in 16 metagenomes, and assembled 13.1 Gbp *de novo* microbial contigs, identifying 22 million non-redundant microbial genes from the cattle rectum microbiome. Fescue toxicosis significantly reduced alpha diversity (*p* < 0.01) and altered beta diversity (PERMANOVA *p* < 0.01), indicating microbial dysbiosis. We discovered significant enrichment of 31 bacterial species post-treatment, including multiple core rumen taxa. *Ruminococcaceae bacterium P7* showed an average of 16-fold increase in fecal abundance (*p* < 0.01), making it the top-featured species in linear discriminant analysis. Functional pathway analysis revealed a shift from energy metabolism to antimicrobial resistance and DNA replication following toxic seed consumption. Comparative analysis showed increased representation of core rumen taxa in rectal microbiota post-treatment, suggesting disrupted rumen function. These findings demonstrate that fescue toxicosis alters both the composition and functional landscape of the hindgut microbiota. *Ruminococcaceae bacterium P7* emerges as a promising biomarker for monitoring fescue toxicosis through non-invasive fecal sampling, with potential applications in herd-level diagnostics and mitigation strategies.

## Simple Summary

This study investigated how fescue toxicosis, a condition caused by cattle consuming endophyte-infected tall fescue, impacts the gut microbiome of beef cattle. By analyzing fecal samples from pregnant cows before and after a 30-day exposure to toxic fescue seeds, we identified significant shifts in their gut bacterial flora using advanced DNA sequencing approaches. Notably, *Ruminococcaceae bacterium P7*, a species normally native to the rumen, increased over 16-fold, emerging as a strong indicator of this disease condition. The study also noted an increase in bacteria associated with antibiotic resistance and cellular repair mechanisms. This research highlights the potential for using non-invasive fecal analysis to monitor key bacterial indicators, leading to better detection and management of fescue toxicosis.

## 1. Introduction

Tall fescue, *Lolium arundinaceum* (*Schreb.*) *Darbysh* is a widely adapted, cool-season perennial grass grown on over 14 million hectares in the Southeastern United States alone [1], feeding over 12 million beef cattle [2]. Most tall fescue plants are infected with an endophyte, *Epichloë coenophiala* [3], which lives in a mutualistic symbiosis with the plant. This mutualism produces secondary metabolites, such as ergot alkaloids, that protect the plant against abiotic and biotic stress [4,5,6,7,8] and increases stand longevity [9]. However, ergot alkaloids (i.e., ergovaline) cause reduced growth and reproductive problems in cattle grazing endophyte-infected tall fescue, which are known as fescue toxicosis [10,11,12]. The economic losses due to fescue toxicosis to the forage-based livestock industry are estimated to be close to USD 3.5 billion each year [13].

The negative effect of tall fescue feeding on cattle growth is affected by the host genotype. Genomic approaches have great potential to identify and select resistant animals. Several studies have investigated genetic markers with a significant contribution to fescue toxicosis tolerance. Among these, a polymorphism within the XK-related 4 gene was discovered to associate with serum prolactin levels [14], and an intronic SNP (single nucleotide polymorphism) was identified in the dopamine receptor D2 gene [15] in beef cows grazing endophyte-infected tall fescue.

The host metabolism is usually significantly affected by fescue toxicosis. Previous studies detected a significant tryptophan and lipid metabolism disruption [16] and dramatic shifts in amino acid metabolism in the plasma [17], among the main consequences of ergovaline consumption. In ruminants, the fermentation is dependent mostly on the rumen microbiota, and the intestinal microbes to a lesser extent [18,19]. The gut microbiome is directly related to important biological functions such as nutrition and digestive health [20,21], affecting almost every aspect of animal physiology, including metabolism, immunity, inflammation, and behavior [22]. The rumen microbiome has been studied extensively [23,24,25,26,27,28,29,30,31,32], and the impact of fescue toxicosis on the rumen microbiome was previously reported, summarizing the metabolic and physiologic impact on cattle and discussing different mitigation strategies [33]. However, the fecal/rectum microbiota and its response to ergovaline are not well understood, with only 16S rDNA gene sequencing studies [17,34,35].

To address this knowledge gap, we conducted whole-genome shotgun (WGS) metagenomic analyses of the rectum microbiome in beef cattle before and after supplementation with endophyte-infected tall fescue seeds. Fecal samples were chosen due to the practicality and non-invasive nature of sample collection in field settings, making them advantageous for developing microbiome-based biomarkers of fescue toxicosis. While previous studies using 16S rRNA ampliconic sequencing have demonstrated that toxic fescue affects fecal microbiome diversity [17,34,35], their taxonomic resolution is limited primarily to the genus level. Animal gut microbiota is extremely complex, with more than 1000 species of microbes [36]. Beyond the taxonomy composition, extensive horizontal gene transfer (HGT) [37] and copy number variation at the species and strain level add additional layers of complexity [38]. Thus, short-read amplicons (~300 bp) used in the 16S rDNA gene sequencing approach lack finer resolution into the functional genomic complexity inherent in microbiomes. In contrast, WGS metagenomic sequencing allows species- and strain-level resolution, quantification of abundance shifts within species, and detection of enriched metabolic pathways and gene networks [39]. Our approaches in this study provide a more comprehensive view of microbiome alterations in response to fescue toxicosis in beef cattle.

## 2. Materials and Methods

### 2.1. Animal Selection and Maintenance

**Figure 1:**
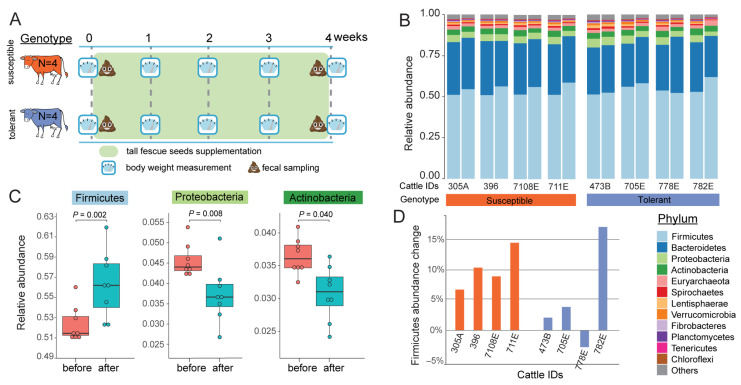
**Fescue toxicosis alters phylum-level composition of the cattle rectum microbiome.** (**A**) Schematic illustration of the experimental design. Eight pregnant cows and heifers were classified as susceptible (n = 4, orange) or tolerant (n = 4, blue) based on genetic testing. All animals were supplemented with toxic tall fescue seeds for 30 days. Body weight was recorded weekly, and fecal samples were collected before (week 0) and after (week 4) the supplementation period. (**B**) Stacked bar plot of relative phylum-level abundance before (***left*** bar) and after (***right*** bar) treatment in individual animals. (**C**) Boxplots showing significant phylum-level shifts in microbial composition after fescue treatment. (**D**) Individual-level changes in Firmicutes abundance after treatment across all animals.

Eight Angus × Simmental pregnant cows (n = 3) and heifers (n = 5) from Alabama Agricultural Experiment Station Black Belt Research and Extension Center (Marion Junction, AL, USA) were selected for tall fescue toxicosis analysis (Figure 1A). Based on the fescue toxicosis tolerance index (Table S1), animals were assigned to two groups: susceptible (n = 4; two cows and two heifers) and tolerant (n = 4; one cow and three heifers). The toxicosis tolerance indices were determined based on a genetic test for tolerance to fescue toxicosis (Agbotanica, LLC, Columbia, MO, USA) [40,41,42], which provides a tolerance index ranging from zero or one star (most susceptible) to four or five stars (most tolerant). After the animals were selected, they were transported to the Beef Evaluation Center at Auburn University, Auburn, Alabama. Before the administration of tall fescue seeds, animals were successfully adapted to the Calan Gate System (Northwood, NH, USA), used for better control of individual dry matter intake. All procedures were approved by the Auburn University Animal Care and Use Committee (IACUC; PRN# 2019-3484).

### 2.2. Endophyte-Infected Tall Fescue Seed Feed Supplement

The tolerant and susceptible animal groups have uniform average body weight (BW; 562 ± 14 kg; *p* = 0.69) within groups. To ensure a daily dose of ergovaline that produces specific signs of fescue toxicosis in beef cattle, we formulated the diet to achieve an individual daily intake of 20 µg of ergovaline/kg body weight/day. Specifically, each animal of both groups received treatment twice per day, and all animals included received the following diet: 2.55 kg of endophyte-infected Kentucky 31 1 tall fescue seeds, 1.16 kg of soybean hulls, 1.16 kg of corn gluten feed, and 0.24 kg molasses on a dry matter basis in addition to *ad libitum* Bermudagrass hay. The ergovaline content in fescue seeds was 5000 ppb on a DM basis, as measured by high-performance liquid chromatography (HPLC; detection limit = 50 ppb and CV = 7%) [43] at the Veterinary Medical Diagnostic Laboratory of the University of Missouri. Animals had free access to water and mineral supplementation blocks (Big 6^®^ Mineral Salt; American Stockman, Overland Park, KS, USA) during the adaptation period and the trial. The study started when the animals were on average six months pregnant and received Kentucky 31 fescue seeds for 30 days. The duration of the feeding period was selected based on previous studies by us and others showing that a 30-day exposure is sufficient to elicit measurable physiological and metabolic negative impacts associated with fescue toxicosis in cattle consuming endophyte-infected tall fescue [17,44]. Individual body weight was obtained on a weekly basis prior to morning feeding (Table S2).

### 2.3. Fecal Sample Collection, Microbial DNA Extraction, and Metagenomic Sequencing

Fecal samples were obtained on day 0 (1 July 2019) and day 29 (29 July 2019) of the trial using single-use fecal loops (Henry Schein^®^, Melville, NY, USA) and immediately placed on liquid nitrogen for posterior storage in −80 °C freezer until laboratory analysis. Fecal DNA samples were extracted using the Qiagen Allprep PowerFecal DNA/RNA kit (Qiagen, Germantown, MD, USA). The homogenization step was conducted using the Qiagen PowerLyzer24 instrument (Qiagen, Germantown, MD, USA). The DNA concentrations were measured by Qubit fluorometer (Invitrogen, Carlsbad, CA, USA) with dsDNA high-sensitive assay kit. A total of 2 μg of genomic DNA was fragmented by M220 Focused-ultrasonicator with a 500 bp targeted insert size (Covaris, Woburn, MA, USA). Metagenomic sequencing libraries were made using the NEBNext Ultra II DNA Library Prep Kit for Illumina (New England Biolabs, Ipswich, MA, USA), following the manufacturer’s protocol. The size range of the final library insert size was 500–600 bp. The libraries were sequenced on an Illumina NovaSeq6000 sequencing machine with 150-bp paired-end reads at the Genomics Service Laboratory at the HudsonAlpha Institute for Biotechnology (Huntsville, AL, USA).

### 2.4. Metagenomic Data Quality Control and Preprocessing

On average, we generated 10.1 billion base-pairs (bp) of sequences per sample for each of the 16 metagenomes (Table S3). The metagenomic reads were quality checked using FastQC (v0.11.9) [44]. The paired-end reads were trimmed to remove Illumina adapter sequences and low-quality bases by Trimmomatic (v0.36) [45]. High-quality filtered reads were mapped to the cattle reference genome (GenBank: GCA_002263795.2) using Burrows-Wheeler Aligner (BWA) (v0.7.17-r1188) [46], to remove any host contaminations (Table S3). The surviving reads were aligned to the viral genome database downloaded from the National Center for Biotechnology Information (NCBI) to remove the viral sequences (Table S3) [47]. The bacterial reads were extracted using SAMtools (v1.6) [48,49] and BEDTools (v2.25.0) [50] for subsequent analysis.

### 2.5. Metagenome Contig Assembly, Taxonomy and Microbial Gene Annotation

To obtain high-quality *de novo* assembly, after the quality control pipeline, we joined the paired-end (PE) reads using PEAR (v0.9.11) [51] to obtain longer merged reads. We assembled the merged single-end reads and PE reads into metagenomic contigs using MEGAHIT (v1.1.2) [52] based on the de Bruijn graph [53] approach. Redundant contigs were removed through clustering using CD-HIT (v4.7) [54] with a 97% sequence identity threshold. Taxonomy assignments for these non-redundant metagenomic contigs were performed using Kaiju (v1.7.3) [55] at species, genus, family, order, class, and phylum levels. Microbial genes were predicted from assembled non-redundant contigs using meta-gene predictor MetaGeneMark (v3.38) [56,57].

### 2.6. Microbial Diversity and Relative Abundance Analyses

**Figure 2:**
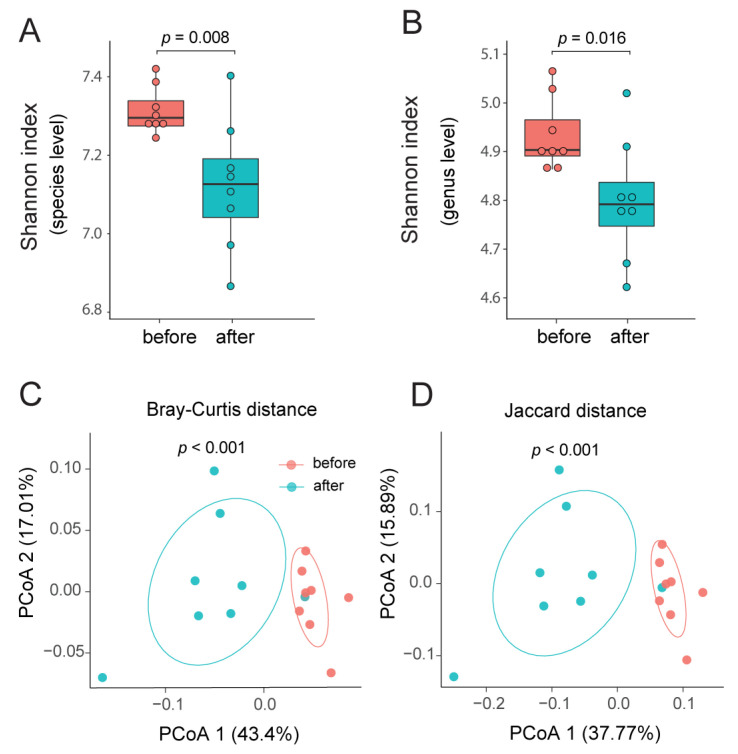
**Tall fescue toxicosis reduces microbial diversity and alters rectum microbiome composition.** (**A**,**B**) Boxplots of alpha diversity before (***red***) and after (***blue***) treatment at the species level (**A**) and genus level (**B**), measured using the Shannon index. Statistical significance was assessed by a two-sided Wilcoxon signed-rank test. (**C**,**D**) The Principal Coordinates Analysis (PCoA) plots of beta diversity between before- and after-treatment rectum microbiome using Bray–Curtis distance (**C**) and Jaccard distance (**D**).

The high-quality filtered PE reads from each metagenome were aligned to the rectum metagenomic contigs we assembled to generate mapping count tables (Table S4). With the counts tables and contig taxonomy annotation, we computed the relative taxonomy frequency using custom Python (v3.7.7) scripts (Data S1–S4; Tables S5 and S6). The relative frequency bar-plots were generated using the R package ggplot2 v3.3.3 [58] and reshape2 v4.0.5 [59]. To assess the statistical significance of the differential abundance, two-sided Wilcoxon signed-rank tests were performed in R using the package ggpubr (Tables S7 and S8). The heatmap plots were generated using R packages pheatmap (v1.0.12) and grid (v4.0.5). The *q*-values were calculated using the *q*-value (v2.22.0) package in R. Alpha-diversity was analyzed using the R package vegan v2.5.7 [60] with Shannon index [61]. The beta-diversity was analyzed based on the Bray–Curtis dissimilarity [62] and Jaccard index in R. The Principal Coordinates Analysis (PCoA) was performed in R [63] (Figure 2).

### 2.7. Linear Discriminant Analysis of the Microbiome Before and After Toxic Seed Consumption

**Figure 3:**
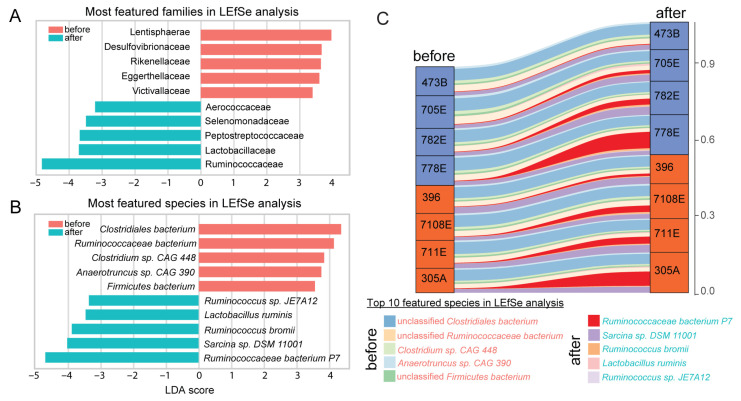
**Featured microbial families and species differentiating rectum microbiome before and after tall fescue toxicosis identified by LEfSe analysis.** (**A**,**B**) The linear discriminant analysis (LDA) scores of the top 10 most featured families (**A**) and species (**B**) in cattle rectum microbiota before and after tall fescue seed treatment (***red***: enriched before treatment; ***blue***: after treatment). (**C**) Alluvial plot illustrating abundance shifts of the top 10 featured species between before and after treatment across eight individual animals. Animal IDs are shown along the *y*-axis, and are color-coded based on the T-snip genotypes (***orange***: susceptible; ***purple***: tolerant). Line width represents relative abundance, and line colors correspond to the individual microbial species.

We performed linear discriminant analysis Effect Size (LEfSe v1.1.1) analysis to determine the most relevant features that explain the differences between groups [64] (Figure 3). Relative taxonomy composition frequencies were used to determine the characteristic microbial taxa in the LEfSe pipeline. HUMAnN2, a pipeline for profiling the abundance of microbial pathways [65], was used to generate the functional pathway profiles in the 16 metagenomes. The most featured functional pathways were identified based on HUMAnN2 output using LEfSe. The alluvial plot was generated using the R package ggalluvial v0.12.3 [66].

### 2.8. Ruminococcaceae bacterium P7 Relative Abundance Analysis and qPCR Validation

Changes in the relative abundance of *Ruminococcaceae bacterium P7* before and after toxic fescue treatment were visualized using bar plots and heatmaps. Hierarchical phylogenetic analysis, spanning from species to phylum level, was performed based on count tables derived from metagenomic read mapping. To visualize both phylogenetic relationships and proportional abundance, we employed Krona (v2.8) [67], focusing on the placement of *Ruminococcaceae bacterium P7* within the Firmicutes phylum and the Ruminococcaceae family. Additionally, we compared the relative abundance profiles of 17 Ruminococcaceae species before and after treatment using a heatmap to evaluate species-specific responses within the family.

To validate the *Ruminococcaceae bacterium P7* abundance changes detected in the metagenomic data, we designed primers for quantitative PCR experiments to target two single-copy genes, *rnhB* and *recR*. The qPCR primers were synthesized by Eurofins (Eurofins Genomics Inc., Huntsville, AL, USA). The qPCR experiments were performed using SYBR Green (Cat No. A25776, Thermo Fisher Scientific, Waltham, MA, USA) in 96-well plates on a Bio-Rad C1000 Touch Thermal Cycler with CFX96 Real-Time PCR Detection Systems (Bio-Rad Laboratories, Hercules, CA, USA). Initial analysis was done using the CFX Maestro software version 2.0. Metagenomic contig cattle_contig_000004026845 (annotated as *Butyrivibrio* sp. *AE2032*) was selected as the control because stable relative abundance was observed across all 16 metagenomes. Relative quantification was performed based on ratios of *Ruminococcaceae bacterium P7* and control species abundance. Two technical replicates were included for each sample.

### 2.9. Comparative Analysis of Rectum and Rumen Microbiome Using Rumen Reference Metagenomes

To evaluate the similarity between the rectum microbiome composition observed in this study and that of the rumen microbiome, we compared our metagenomic data with a high-quality cattle rumen reference metagenome assembled from 4941 individual rumen metagenomes (European Nucleotide Archive accession: PRJEB31266). The reference genome, along with the top 41 core rumen microbial genera and top 31 abundant rumen microbial families, was adopted from Stewart et al. [68]. Paired-end reads from our rectum samples were aligned to the rumen reference to quantify shared microbial composition. Venn diagrams illustrating the overlap between the rectum and rumen microbiomes were generated using Python packages matplotlib (v3.3.4) [69] and matplotlib_venn (v0.11.6). Relative abundances of microbial taxa were calculated based on counts per million (CPM) of mapped reads, using custom Python scripts [70].

### 2.10. Functional Pathway Profiling and KEGG-Based Enrichment Analysis

Functional profiling of all rectum microbiome samples was performed using the HUMAnN2 pipeline [66], which quantifies gene families and metabolic pathways from metagenomic data. Pathway annotations were conducted using the Kyoto Encyclopedia of Genes and Genomes (KEGG) database [71]. To identify differentially abundant functional features between pre- and post-treatment groups, we applied Linear Discriminant Analysis Effect Size (LEfSe) to KEGG Ortholog tables, using normalized counts per million (CPM) for relative abundance estimation. KEGG Orthologs were further organized into KEGG BRITE hierarchies to facilitate interpretation of functional enrichment at higher-order biological categories.

### 2.11. Antimicrobial Resistome Analysis Pre- and Post-Tall Fescue Treatment

We used ABRicate (v0.8.13) to mass screen our rectum microbial contigs for antimicrobial resistance genes (ARGs) using the Resfinder database [72]. The ARGs were further grouped into ARG families using the Comprehensive Antibiotic Resistance Database (CARD) [73]. We extracted the reads aligned to the annotated ARGs to quantify the relative abundance of each ARG family with custom Python scripts (Data S5).

## 3. Results

### 3.1. A Comprehensive Assembly of Cattle Rectum Microbiome Using WGS Metagenomic Data

We generated a total of 157 Gbp of WGS metagenomic sequences from 16 fecal samples representing the cattle rectum microbiome. Each sample yielded an average of 65.4 million 150 bp PE reads (Figure 1A; Table S3). After removing low-quality reads (2.20%), host-derived sequences (6.31%), and viral reads (0.03%), 104.4 Gbp of high-quality microbial reads remained and were used for downstream analyses. The *de novo* assembly of these reads resulted in a non-redundant rectum microbial contig reference comprising 16,580,560 contigs, ranging in length from 400 bp to 406,369 bp, with an N50 of 787 bp and a total assembly size of 13.1 Gbp. Among the rectum reference microbial contigs we assembled, 70.0% were assigned to a specific phylum, and 50.3% have species-level annotations. Alignment of metagenomic reads to the assembled reference yielded an average mapping rate of 91.7% (Table S4), indicating high assembly completeness. Microbial gene prediction on the assembled contigs identified 21,950,894 non-redundant genes, with a combined gene sequence length of 9.8 Gbp and an N50 of 540 bp. This dataset provides a comprehensive genomic resource for investigating microbial dynamics in the beef cattle gut microbiome.

### 3.2. Fescue Toxicosis Induces Phylum-Level Shifts with Firmicutes Enrichment in Gut Microbiome

The most abundant phyla in cattle rectum microbiota were Firmicutes and Bacteroidetes (Figure 1B), together accounting for ~80% of the total abundance, consistent with previous studies using 16S rDNA data [35]. Following the 30-day toxic fescue seed supplementation, Firmicutes exhibited a significant increase of 7.6% (*p* = 0.002), while Proteobacteria and Actinobacteria showed significant reductions of 18.1% (*p* = 0.008) and 15.6% (*p* = 0.04), respectively (Figure 1C). These phylum-level shifts suggest a pronounced microbial response to toxic fescue exposure.

We examined if there was a difference in phylum-level compositional change according to the T-snip genotypes. A trend toward a greater increase in Firmicutes abundance after treatment was observed in susceptible genotypes, with an average increase of 10.1%, compared to 5.1% in tolerant individuals. However, this difference was not statistically significant (*p* = 0.31, Mann–Whitney U test; Figure 1D).

### 3.3. Reduced Microbiome Diversity Following Tall Fescue Seed Supplementation

After the 30-day consumption of endophyte-infected tall fescue seed, the microbial alpha diversities in the rectum microbiome declined significantly at both species-level (Figure 2A, *p* = 0.008, Wilcoxon signed-rank test) and genus-level (Figure 2B, *p* = 0.016). Our results indicate a strong reduction in rectum microbiome complexity after tall fescue seed treatment. Beta diversities were analyzed and plotted for the rectum metagenomes before and after treatment. Principal coordinates analysis (PCoA) identified significant separation for the two groups under both the Bray–Curtis dissimilarity measurement (Figure 2C, *p* = 0.002, PERMANOVA, Permutational multivariate analysis of variance) and Jaccard distance (Figure 2D, *p* = 0.004, PERMANOVA). Collectively, these results revealed that toxic fescue exposure induces significant shifts in the composition and diversity of the rectum microbiome, indicating potential microbial dysbiosis.

### 3.4. Top 10 Discriminative Families and Species After Tall Fescue Seed Supplementation

To identify the microbial families and species that changed most significantly, we performed Linear Discriminant Analysis (LDA) to determine the most featured microbial taxa before and after toxic tall fescue treatment. Families significantly enriched after treatment (LDA score cutoff of 3.0) included Ruminococcaceae (LDA score = 4.67), Lactobacillaceae (3.62), Peptostreptococcaceae (3.55), Selenomonadaceae (3.37), and Aerococcaceae (3.10). In contrast, Lentisphaerae (3.81), Desulfovibrionaceae (3.56), Rikenellaceae (3.53), Eggerthellaceae (3.48), and Victivallaceae (3.30) were significantly reduced after treatment (Figure 3A, *p* < 0.05, Wilcoxon signed-rank test).

At the species level (Figure 3B), the top five enriched species after treatment were *Ruminococcaceae bacterium P7* (16-fold increase), *Sarcina* sp. *DSM 11,001* (48.5% increase), *Ruminococcus bromii* (3-fold increase), *Lactobacillus ruminis* (14-fold), and Ruminococcus sp. JE7A12 P7 (6-fold). Among the five most decreased species, only *Clostridium* sp. *CAG 448* and *Anaerotruncus* sp. *CAG 390* had specific annotations, and the remaining three were unclassified Firmicutes, Ruminococcaceae, or Clostridiales (Figure 3B).

An alluvial plot of the compositional change revealed a sharp contrast in abundance shifts: the top five enriched species showed a 215.8% overall increase, while the top five decreased species only exhibited a 15.2% reduction (Figure 3C). This asymmetry was consistent when all significantly enriched and depleted species were analyzed (Figure S1), further highlighting the cattle microbiome’s disproportionate response to toxic fescue exposure.

### 3.5. Species-Specific Response of Ruminococcaceae bacterium P7 Is a Hallmark of Fescue Toxicosis

**Figure 4:**
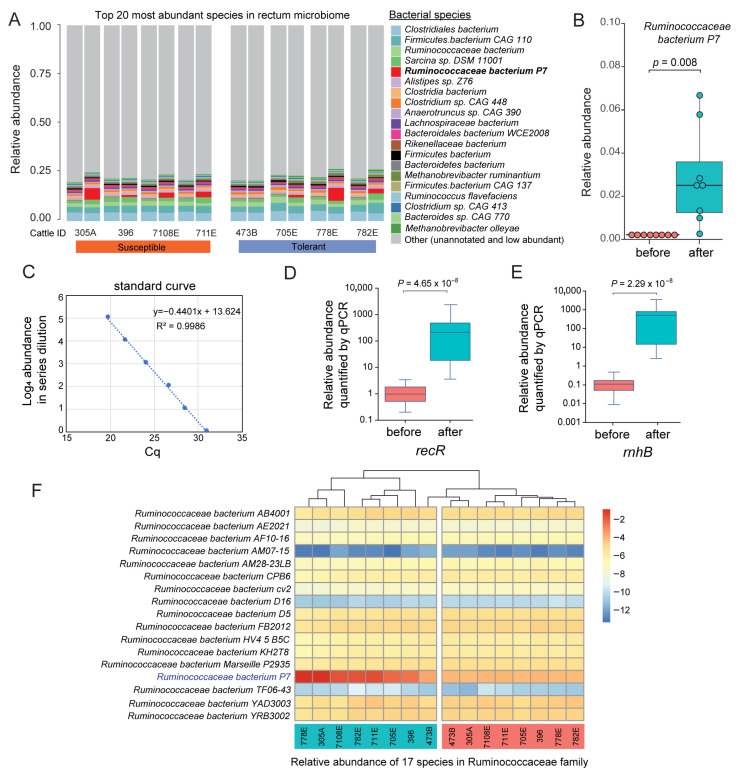
***Ruminococcaceae P7* is selectively enriched in the rectum microbiome under fescue toxicosis and validated by qPCR.** (**A**) Stacked barplot showing the relative abundance of the top 20 most abundant bacterial species in the rectum microbiome across eight animals before (***left*** bar) and after (***right*** bar) tall fescue seed treatment. The less abundant and unannotated species were merged into the “Other” group shown in grey. (**B**) Boxplot of *Ruminococcaceae bacterium P7* relative abundance before and after treatment based on metagenomic data (*p* = 0.008, Wilcoxon signed-rank test). (**C**) Standard curve used for quantitative PCR (qPCR) validation, showing the relationship between Cq values and log-transformed abundance (R^2^ = 0.9986). (**D**,**E**) Box plots of qPCR quantification for two single-copy genes (*recR* and *rnhB*) in the *Ruminococcaceae bacterium P7* genome, both confirming significant enrichment after treatment (*p* < 0.001). (**F**) Heatmap of the relative abundance of 17 Ruminococcaceae species with detectable abundance in the rectum microbiome across individual animals before and after treatment.

*Ruminococcaceae bacterium P7* emerged as the most distinctive species differentiating the rectum microbiome before and after toxic fescue treatment, and it is also one of the top 20 most abundant microbial species (Figure 4A). At the species level, it exhibited a striking 16-fold increase in relative abundance post-treatment (*p* = 0.0078; Figure 4B), a pattern consistently observed across all eight animals (Figure 3C and Figure 4A). This result was further validated by qPCR using two independent genes in its genome (Figure 4C–E). *Ruminococcaceae bacterium P7* is named based on its family-level classification, as it lacks genus-level annotation (unclassified Ruminococcaceae, NCBI Taxonomy ID 1200751). The best available NCBI reference genome (accession GCF_900100595.1) was suppressed due to contamination concerns by NCBI curators. To confirm the presence of this species in the cattle microbiome, we reconstructed its genome as a metagenome-assembled genome (MAG) from our metagenomic data. The resulting assembly yielded a 2.3 Mbp genome containing 2125 annotated coding genes. The checkM-assessed the genome completeness as 100% with a level of contamination of 12.07%. To further investigate the specificity of the microbial response within the Ruminococcaceae family, we examined species-level abundance patterns of 17 Ruminococcaceae species to determine whether the observed increase was representative of a broader family-level response. Notably, *Ruminococcaceae bacterium P7* was the only species within this family to exhibit a significant increase following tall fescue treatment (Figure 4F), indicating a distinct, species-specific response to fescue toxicosis.

### 3.6. Post-Treatment Firmicutes Enrichment Is Primarily Driven by Ruminococcaceae bacterium P7

**Figure 5:**
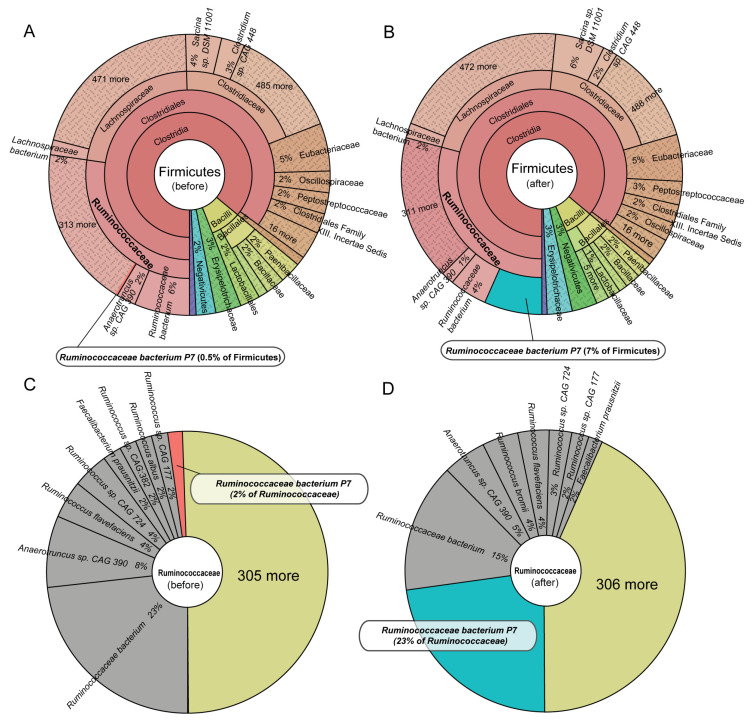
***Ruminococcaceae bacterium P7* drives Firmicutes enrichment in the rectum microbiota following tall fescue toxicosis.** (**A**,**B**) Krona plots visualizing the taxonomic composition of Firmicutes in the rectum microbiome before (**A**) and after (**B**) toxic fescue consumption. Distinct taxonomic groups are color-coded, with composition percentages labeled at the family level. The size of each section is proportional to its relative abundance in the microbiome. (**C**,**D**) Pie charts displaying species-level composition within the Ruminococcaceae family before (**C**) and after (**D**) treatment.

In the cattle rectum microbiome, ~50% of microbes belong to the Firmicutes phylum (Figure 1B), with Ruminococcaceae identified as the most abundant family (Figure S2), suggesting its potential functional importance in the gut. To determine whether the observed post-treatment enrichment of Firmicutes was a broad phylum-level effect or driven by specific taxa, we used Krona plots to visualize the hierarchical taxonomic composition before and after toxic fescue consumption. The Krona visualizations revealed that the enrichment of Firmicutes after treatment was not uniformly distributed across this phylum but was primarily driven by a sharp increase in the abundance of the *Ruminococcaceae bacterium P7*. Prior to treatment, this species accounted for only 0.5% of total Firmicutes, and its relative abundance increased to 7% post-treatment (Figure 5A,B). When focusing on the Ruminococcaceae family, *Ruminococcaceae bacterium P7* increased from 2% to 23% of the family-level composition (Figure 5C,D), representing the most pronounced species-specific change. In contrast, the abundance of other major family members, such as *Ruminococcus flavefaciens* and *Anaerotruncus* sp. *CAG 390* remained relatively stable. These findings suggest that the overall increase in Firmicutes after fescue supplementation is largely attributable to the dramatic and selective expansion of the *Ruminococcaceae bacterium P7*.

### 3.7. Significant Increase of Rumen-Associated Microbes in Rectum Microbiome Under Fescue Toxicosis

Ruminococcaceae is a core family within the rumen microbiome, and the observed increase in *Ruminococcaceae bacterium P7* in the rectum may reflect a microbial shift or translocation from the rumen to the hindgut in response to tall fescue toxicosis. To investigate the differences in microbial composition and abundance between the rectum and rumen microbiome, we aligned our metagenomic reads to the rumen reference metagenomes from Stewart et al. [68]. A total of 34.0% rectum microbial reads were mapped to the rumen reference, indicating that over one-third of the rectum microbiome could also be found in the rumen. Following the 30-day toxic fescue seed supplementation, there was a 10.1% increase in the proportion of rumen-mapped reads in the rectum samples, a change that is marginally significant (*p* = 0.078). These results suggest a trend toward increased representation of rumen-origin microbes in the rectum microbiome after treatment.

**Figure 6:**
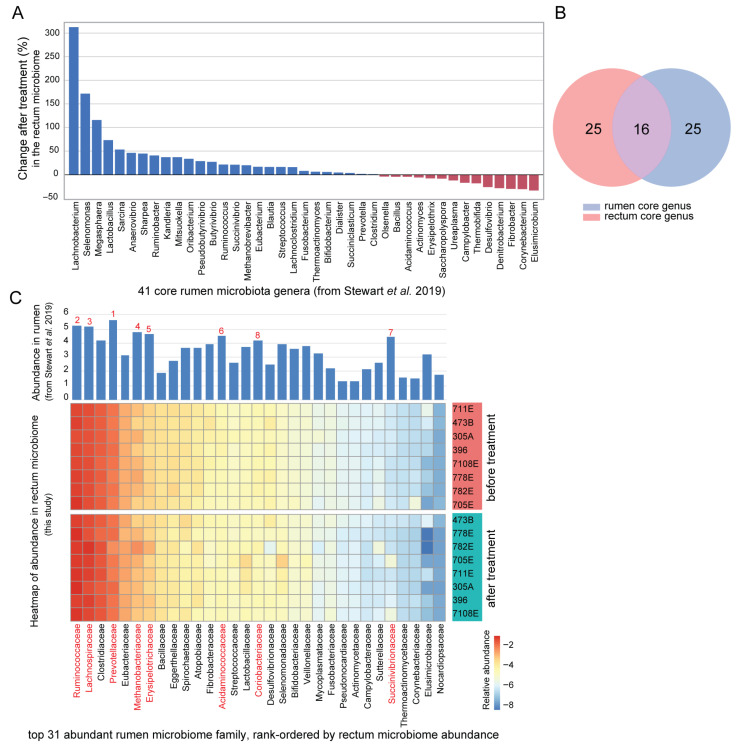
**Core rumen microbiome taxa are enriched in the rectum microbiome following tall fescue toxicosis.** (**A**) Barplot showing percent change in abundance of 41 core rumen microbial genera in the rectum microbiome after toxic fescue treatment. (**B**) Venn diagram illustrating the overlap between core rumen and rectum microbial genera. (**C**) Heatmap of the top 31 abundant rumen microbial families (from Stewart et al., 2019 [68]), ranked by their abundance in the rectum microbiome. The bar plot (***top***) shows their corresponding abundance in the rumen. The heatmap displays relative abundance in the rectum microbiome before (***top***) and after (***bottom***) toxic fescue treatment. The eight core microbial families in the rumen microbiome are labeled in red.

To further examine this observation in detail, we quantified the abundance of 41 core rumen genera [69] in our rectum data, and identified that over 65% (27/41) of the genera showed increased abundance, with notable enrichment in genera such as *Lachnobacterium*, *Ruminococcus*, *Selenomonas*, and *Megasphaera* (Figure 6A). Of the 41 core genera, 16 are shared between the two communities, while 25 are unique to each (Figure 6B). Because Ruminococcaceae bacterium P7 lacks genus-level annotation, we compared the abundance profiles of the top 31 rumen microbial families reported by Stewart et al. [68], evaluating their corresponding abundance in the rectum microbiota before and after treatment. Overall, families that were highly abundant in the rumen also tended to rank highly in the rectum microbiome (Figure 6C). Six of the top eight core rumen families were also among the most abundant in the rectum, including Ruminococcaceae, Lachnospiraceae, Prevotellaceae, Methanobacteriaceae, Erysipelotrichaceae, and Acidaminococcaceae (Figure 6C). This overlap indicated potential functional importance of these families to both the rumen and the hindgut. Notably, Ruminococcaceae was the only family among these that significantly distinguished the microbiome before and after treatment, highlighting its potential role in the microbial response to fescue toxicosis.

### 3.8. Functional Shifts in the Rectum Microbiome Reveal Increased Antimicrobial Resistance and Decreased Energy Metabolism Following Fescue Toxicosis

**Figure 7:**
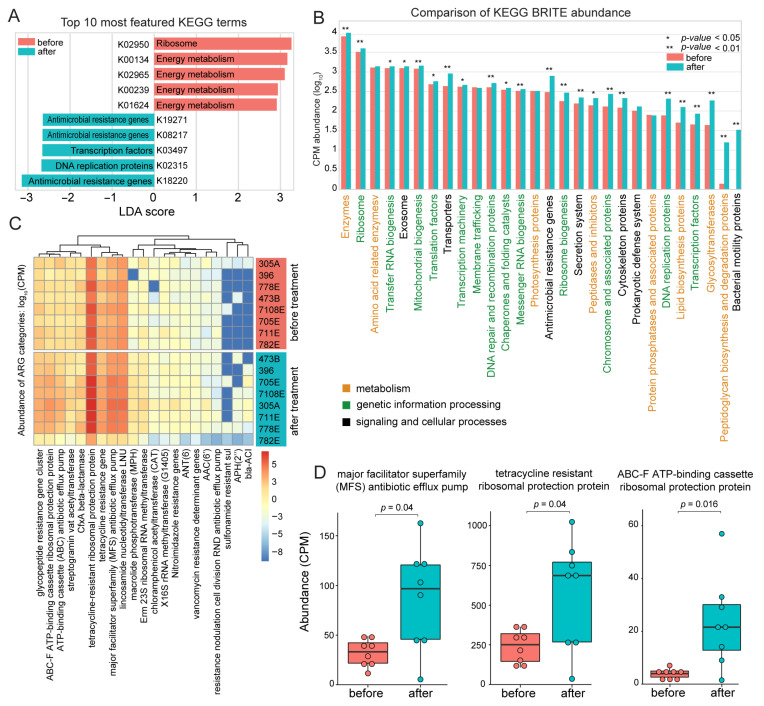
**Functional enrichment of antimicrobial resistance and DNA replication pathways in the rectum microbiome following fescue toxicosis.** (**A**) Linear discriminant analysis (LDA) of KEGG orthologs identified the top 10 most featured functional terms between pre- and post-treatment groups. (**B**) Barplot showing significant differential abundance of KEGG BRITE functional categories pre- and post-treatment (*, *p* < 0.05; **, *p* < 0.01, Wilcoxon signed-rank test), measured by counts per million (CPM). KEGG BRITE term labels are color-coded according to their functional category: metabolism (orange), genetic information processing (green), and cellular processes (black). (**C**) Heatmap of antimicrobial resistance gene (ARG) family abundance across individual animals before and after treatment. (**D**) Boxplots showing significantly increased abundance of three major ARG classes post-treatment. Statistical significance was assessed using the Wilcoxon signed-rank test.

Linear discriminant analysis (LDA) identified the top 10 KEGG orthologs that most strongly distinguished the rectum microbiome before and after toxic fescue treatment. Notably, orthologs related to energy metabolism were significantly overrepresented in pre-treatment samples, while antimicrobial resistance genes (ARGs), DNA replication proteins, and transcription factors were enriched post-treatment (Figure 7A). To explore broader functional changes, we grouped all annotated KEGG orthologs into KEGG BRITE hierarchical categories. Of the 28 BRITE categories examined, 17 were significantly more abundant after treatment (*p* < 0.05, Wilcoxon signed-rank test; Figure 7B). Eight of these categories showed more than a two-fold increase in abundance, including transporters, ARGs, chromosome-associated proteins, DNA replication proteins, lipid biosynthesis proteins, glycosyltransferases, peptidoglycan biosynthesis and degradation proteins, and bacterial motility proteins (Figure 7B).

To further characterize antimicrobial resistance, we profiled the resistome of the rectum microbiome by quantifying ARGs in pre- and post-treatment samples (see Methods). A total of 89 ARGs were identified before treatment and 94 after, indicating an increase in ARG diversity under fescue toxicosis. These ARGs were grouped into 21 functional categories, and their relative abundance was quantified for each individual metagenome (Figure 7C). Three ARG families showed significant enrichment following treatment: tetracycline-resistant ribosomal protection proteins, major facilitator superfamily (MFS) antibiotic efflux pumps, and ABC-F ATP-binding cassette ribosomal protection proteins (*p* < 0.05; Wilcoxon signed-rank test; Figure 7D). These findings indicate an increase in the antimicrobial resistance potential of the gut microbiome in response to toxic fescue exposure.

## 4. Discussion

### 4.1. Comprehensive Profiling of the Cattle Rectal/Fecal Microbiome Using WGS Metagenomics

To improve the feed efficiency and reduce greenhouse gas emissions, cattle rumen microbiota have been extensively studied at the WGS metagenomic level [31,32], metatranscriptomic level [28,29], as well as individual genome sequencing of rumen microbes [30,74]. In contrast, there is a paucity of rectal/fecal microbiome studies using a WGS metagenomic approach. In our study, we sequenced and assembled the rectum metagenome from 16 individual samples, identifying 22 million non-redundant microbial genes. The cattle rectum microbiome appears to be more complex than that of humans [75], rodents [76,77], dogs [78], or pigs [79]. Notably, 34% of the rectum microbial sequences could be mapped to the rumen metagenomes. A previous study using 16S rDNA amplicon sequencing in Nelore cattle found that fecal/rectal microbiome OTUs closely resemble those of the cecum and colon, suggesting that rectal samples may reflect microbial composition of the large intestine [74]. The most dominant phyla in our dataset were Firmicutes (54.4%) and Bacteroidetes (29.6%), with Ruminococcaceae (13.8%) and Lachnospiraceae (11.7%) being the most abundant families, which are consistent with previous studies using 16S rDNA amplicon analyses [35,74]. Our microbial gene catalog provides a valuable reference for future studies investigating the cattle microbiome using fecal samples.

### 4.2. Physiological Effects of Fescue Toxicosis After Toxic Seed Consumption

In cattle, fescue toxicosis caused by the intake of endophyte-infected cool-season grasses produces a significant decrease in animal production due to metabolic and physiological impairments, such as vasoconstriction, reduction in cellulose digestibility, foot rot, and other symptoms [33,80]. For example, Wilbanks et al. used late-gestation beef cows as a model for identifying metabolic perturbance due to fescue toxicosis [81], and they reported a reduction in body weight in cows exposed to endophyte-infected tall fescue compared with those exposed to endophyte-free tall fescue [81]. Fescue toxicosis also reduces short-chain fatty acid (SCFA) absorption in the rumen, mainly due to a lower feed intake [82]. A meta-analysis by Liebe and White (2018) further demonstrated a negative correlation between dietary ergovaline concentration and average daily gain [83].

In our toxic tall fescue seed supplement experiments, no treatment × time interaction effect on body weight was detected (*p* = 0.13). However, we did observe a trend toward weight loss in animals receiving K31 endophyte-infected tall fescue seeds during the trial (*p* = 0.05). This trend aligns with previous studies demonstrating adverse effects of endophyte-infected tall fescue on cattle performance metrics [83,84].

### 4.3. Fescue Toxicosis Induces Gut Microbiome Dysbiosis

Fescue toxicosis alters the gut microbiome through both digestive and nutritional disruptions. One of its key toxic compounds, ergovaline, is known to block monoamine receptors and impair gut motility, potentially disturbing microbial communities adapted to the gastrointestinal environment [85,86]. The impact of fescue toxicosis on the cattle gut microbiome has been previously investigated using 16S rDNA amplicon sequencing. For example, Mote et al. reported significant alterations in beta-diversity, but not alpha-diversity, in animals grazing toxic tall fescue [17]. In another study, Koester et al. compared the microbiomes of 20 high-tolerance and 20 low-tolerance individuals [35] and discovered a significant reduction in alpha diversity in the low-tolerance group.

In our study, WGS metagenomic analysis revealed a significant reduction in alpha-diversity under fescue toxicosis at both microbial species and genus levels. Additionally, we identified significant shifts in beta-diversity between the pre- and post-treatment groups, confirming the presence of dysbiosis in response to toxic tall fescue exposure. At the phylum level, Firmicutes significantly increased, while Proteobacteria and Actinobacteria significantly declined following treatment. Both *Proteobacteria* and *Actinobacteria* include taxa known to be involved in fiber degradation [87]. The observed reduction in their abundance may reflect a decline in fiber digestion efficiency. This is consistent with findings from Koontz et al. (2015), who reported a decreased passage rate in Holstein steers fed endophyte-infected (E+) tall fescue diets, suggesting impaired fiber utilization under fescue toxicosis [88].

The observed reduction in microbial diversity and altered community structure strongly indicate dysbiosis induced by toxic seed ingestion. Ecologically, reduced alpha diversity may compromise the functional redundancy of the gut microbiome, diminishing its resilience to other environmental stressors. Functionally, a loss in microbial richness could impair fiber fermentation and nutrient extraction efficiency, as key taxa involved in cellulolysis and short-chain fatty acid (SCFA) production may be depleted [89]. Furthermore, reduced diversity has been associated with impaired immune modulation and increased susceptibility to pathogen overgrowth [90]. Thus, the microbial dysbiosis observed under fescue toxicosis likely contributes to the broader physiological and metabolic disturbances seen in affected cattle, potentially exacerbating negative outcomes in growth, digestion, and overall health.

### 4.4. Ruminococcaceae bacterium P7 as a Hallmark Species of Microbiome Change

We used linear discriminant analysis effect size (LEfSe), a widely accepted method for microbiome biomarker discovery [64], to identify microbial features distinguishing pre- and post-treatment samples. *Ruminococcaceae bacterium P7* emerged as the most prominent species, with an LDA score five times higher than the next most significant microbial taxon. The relative abundance of *Ruminococcaceae bacterium P7* increased from less than 2% to approximately 25% of the total Ruminococcaceae family post-treatment, which is an over 16-fold enrichment.

At the family level, Ruminococcaceae was also significantly overrepresented post-treatment and ranked as the top biomarker in the LEfSe analysis. As a core component of the rumen microbiome, Ruminococcaceae is involved in fiber digestion [91], including cellulose and hemicellulose degradation [92] and contributes to butyric acid formation in the non-ruminant gut [93]. Previous 16S rDNA studies have demonstrated that the abundance of various Ruminococcaceae genera correlates with average daily gain and production traits in cattle [94]. By using whole-genome shotgun metagenomics, we achieved species- and strain-level resolution, revealing that the observed increase in Ruminococcaceae was specifically driven by *Ruminococcaceae bacterium P7*. Research on this species is limited; however, Kim et al. identified it as the only taxon enriched under normal conditions relative to heat stress in the rumen, suggesting that its abundance decreases under physiological stress [95]. While *Ruminococcaceae bacterium P7* demonstrated significant and consistent enrichment in response to toxic fescue exposure under controlled experimental conditions, its utility as a biomarker in field settings may be influenced by additional environmental and management variables. Factors such as diet composition variability, grazing behavior, co-occurring stressors, regional environmental microbiome, and individual host variation might introduce considerable variations in *Ruminococcaceae bacterium P7* abundance in addition to fescue toxicosis. Therefore, further validation studies in diverse, real-world animal production systems are necessary to confirm its diagnostic robustness and to refine its sensitivity and specificity across different farm and pasture conditions.

### 4.5. Other Rumen Core Genera Responding to Fescue Toxicosis

In addition to the dramatic increase of *Ruminococcaceae bacterium P7*, other core rumen taxa also showed notable shifts in abundance following toxic fescue exposure. Among the top 41 most abundant rumen genera, *Lachnobacterium* exhibited an over 300% increase after treatment. This genus includes only a single known species, *Lachnobacterium bovis*, an anaerobic Gram-negative bacterium commonly found in cattle rumen and feces [96]. *L. bovis* was significantly enriched in the rectum microbiome after treatment, suggesting a notable shift in microbial composition. The second most increased core rumen genus was *Selenomonas*, a crescent-shaped Firmicute genus typically present in the rumen and cecum. In the early growth phase, most *Selenomonas* cells exhibit motility via flagella [97]. Sawanon et al. provided preliminary evidence of its involvement in fiber digestion within the rumen [98]. Two *Selenomonas* species, *S. ruminantium* and *Selenomonas* sp. *mPRGC8*, were significantly elevated in abundance in the rectum microbiome after treatment. These findings suggest possible adaptive roles for these species under fescue-induced stress, though further investigation is needed to elucidate their functional contributions.

Our comparative analysis of the rumen microbiome revealed a trend toward an increased presence of rumen-associated microbes in the rectum under fescue toxicosis. This observed microbial shift is likely driven by multiple interrelated mechanisms. Exposure to ergot alkaloids induces systemic physiological stress that may facilitate microbial translocation from the rumen to the hindgut. This is further exacerbated by alterations in the intestinal environment, including shifts in pH, SCFA concentrations, and other metabolites that can create favorable conditions for rumen-associated microbes to colonize the hindgut. Fescue toxicosis also slows gastrointestinal motility, increasing content retention time and promoting microbial spillover downstream. Additionally, immune dysregulation resulting from toxicosis compromises mucosal barrier integrity and diminishes colonization resistance, allowing upstream microbial taxa to proliferate in the rectum. Lastly, impaired nutrient absorption and changes in nutrient availability further reshape the microbial landscape by favoring taxa adapted to these altered conditions. Collectively, these changes contribute to significant disruptions in the composition and diversity of the rectal microbiome in cattle affected by fescue toxicosis.

### 4.6. Functional Shifts Highlight Enrichment of Antimicrobial Resistance

Functional pathway enrichment analysis of the cattle rectum microbiome revealed that, prior to treatment, four out of the five most featured KEGG terms were associated with energy metabolism—typical of a healthy gut environment. However, following exposure to endophyte-infected tall fescue, the dominant functional categories shifted dramatically, with ARGs and DNA replication proteins becoming significantly overrepresented. To our knowledge, this is the first report of antimicrobial resistome alterations in response to fescue toxicosis. ARG profiling further identified three gene families that were significantly enriched after treatment: tetracycline-resistant ribosomal protection proteins, ABC-F ATP-binding cassette ribosomal protection proteins, and major facilitator superfamily (MFS) antibiotic efflux pumps. The first two function through ribosomal protection, while MFS proteins function as secondary active transporters [99]. Additionally, peptidoglycan biosynthesis proteins and bacterial motility proteins also increased significantly post-treatment, suggesting potential roles in bacterial cell wall synthesis and enhanced motility, possibly facilitating microbial relocation from the rumen to the large intestine under fescue-induced stress.

### 4.7. Limitations of This Study

One limitation of this study is the inclusion of both cows and heifers. Ideally, a study involving only heifers would offer a more uniform population and reduce potential confounding variables. However, due to a concurrent study on rumen-protected nutrient supplementation, the number of available heifers was limited, necessitating the inclusion of cows. In the literature, previous research suggests that age has minimal influence on rumen microbiome composition in adult cattle. Liu et al. (2017) examined age-related changes in rumen bacteria and methanogens and reported only limited variation in microbial composition between primiparous and mature dams [100]. Similarly, Ahmad et al. (2022) found only modest age-dependent differences in rumen bacterial communities across life stages in Mongolian cattle [101]. While the inclusion of both cows and heifers may slightly reduce the statistical power, it is unlikely to introduce spurious results or fundamentally alter the conclusions of this study.

Another limitation of the current study is the relatively small sample size within the genotype-based subgroups, consisting of only four tolerant and four susceptible animals per group. Although we observed that tolerant animals exhibited a lower degree of microbial compositional shifts at the phylum level, the difference reached only marginal statistical significance. The limited number of individuals constrains the statistical power to detect meaningful differences, particularly at finer taxonomic resolutions (e.g., genus or species level) or at the level of functional gene pathways. As a result, analyses at these lower taxonomic levels were not pursued. Future studies with larger cohorts will be necessary to validate these findings and facilitate high-resolution characterization of microbial and functional signatures associated with genotype-specific responses to fescue toxicosis. For the MAG-derived *Ruminococcaceae bacterium P7* genome assembly, the 12% contamination estimated by CheckM is nontrivial and warrants cautious interpretation. Given the presence of at least 17 *Ruminococcaceae bacterium* species in the fecal microbiome, a certain level of contamination during metagenomic assembly is expected due to shared genomic regions with high sequence similarity. To ensure the specificity of downstream applications such as PCR primer design, we manually inspected both the depth and evenness of coverage across contigs to confirm the accurate assignment to the *Ruminococcaceae bacterium P7*. Nonetheless, results derived from this metagenome-assembled genome should be interpreted with appropriate caution, especially when extrapolating to functional or taxonomic conclusions.

### 4.8. A Working Hypothesis for the Mechanism of Microbiome Shift in Response to Fescue Toxicosis

Our findings suggest that consumption of endophyte-infected tall fescue seeds impairs the rumen microbiome and results in a global increase of rumen-associated microbes within the rectum microbiome. However, this shift is not merely a passive translocation due to microbial loss in the rumen; rather, it appears to be highly species-specific. Among the enriched taxa, *Ruminococcaceae bacterium P7* exhibited a >16-fold increase in abundance, while no other Ruminococcaceae species showed significant changes. This striking increase may indicate a unique capacity of the *Ruminococcaceae bacterium P7* to re-adapt and proliferate in the large intestine under toxic stress. One possible explanation is that this species, originally resident in the rumen, migrated and thrived in the colon due to ecological pressures induced by fescue toxicosis. In parallel, we observed significant enrichment of ARGs, indicating functional adaptation of the microbiome. While the precise mechanisms remain unclear, our findings point to a complex, dynamic response to toxic stress, involving both taxonomic restructuring and functional reprogramming.

### 4.9. Ruminococcaceae bacterium P7 as a Fecal Biomarker for Early Diagnosis of Fescue Toxicosis

Given its dramatic and consistent increase in response to toxic tall fescue exposure, *Ruminococcaceae bacterium P7* can emerge as a highly promising candidate biomarker for fescue toxicosis. Its biological specificity and practicality for non-invasive sampling make it suitable for field use. Fecal sampling allows for repeated, large-scale data collection without compromising animal welfare. The substantial and uniform fold-change of *Ruminococcaceae bacterium P7* across all individuals in our study supports its sensitivity and specificity as a robust indicator of microbiome perturbation due to fescue toxicosis. Moreover, its abundance allows for quantification of individual responses, enabling severity assessments, identification of tolerant animals, and informed breeding or management decisions. Most importantly, the enrichment of *Ruminococcaceae bacterium P7* in feces precedes the onset of clinical symptoms, enabling early diagnosis and timely intervention. Early detection is crucial for mitigating productivity losses and preventing long-term physiological damage in affected animals. Thus, monitoring through the *Ruminococcaceae bacterium P7* abundance offers a powerful tool for proactive herd management and improving resilience to fescue toxicosis.

## 5. Conclusions

This study presents a comprehensive WGS metagenomic analysis of beef cattle rectum microbiome in response to fescue toxicosis, a condition known to impair growth, reproduction, and overall productivity in beef cattle. We observed a significant reduction in both alpha- and beta-diversity following exposure to toxic tall fescue seeds, indicating a shift toward gut microbial dysbiosis. Notably, we identified a pronounced enrichment of core rumen taxa in the rectum microbiome, with *Ruminococcaceae bacterium P7* emerging as a key species, exhibiting more than a 16-fold increase in abundance. This species-specific response highlights the dynamic nature of the gut microbiome under toxic stress. In addition to taxonomic shifts, functional profiling revealed an unexpected increase in antimicrobial resistance genes, suggesting a broader physiological response to the toxic challenge. These results might not only advance our understanding of the taxonomic and functional disruptions associated with fescue toxicosis but also identify *Ruminococcaceae bacterium P7* as a promising fecal biomarker. Given its specificity, consistency, and feasibility for non-invasive sampling, this species could hold strong potential for early diagnosis, herd health monitoring, and the development of mitigation strategies, including selective breeding for resistance. However, future studies should involve a larger sample size in order to confirm the observed pattern of microbial enrichment under fescue toxicosis. Overall, our findings offer critical insights into the microbial mechanisms underlying fescue toxicosis and lay the groundwork for improved animal health management.

## Data Availability

The whole-genome shotgun metagenomic sequencing data have been deposited in the NCBI Sequence Read Archive (SRA) under accession number PRJNA744219.

## Supplementary Materials

The following supporting information can be downloaded at: https://www.mdpi.com/article/10.3390/biology14091197/s1, Table S1. Genotypic classification of tall fescue toxicosis tolerance using T-snip assay in cattle enrolled for rectal metagenomic profiling. Table S2. Bodyweight trajectories of individual cattle during the 4-week toxic fescue seed feeding experiment. Table S3. Whole-genome shotgun metagenomic sequencing yield and quality control metrics for cattle rectum/fecal samples. Table S4. Read mapping statistics of rectum metagenomes against the assembled microbial reference database. Table S5. Top 20 most abundant bacterial families in the cattle rectum microbiome before and after fescue toxicosis treatment. Table S6. Top 20 most abundant bacterial species identified in the cattle rectum microbiome across treatment conditions. Table S7. Bacterial species significantly enriched in the rectum microbiome after exposure to endophyte-infected tall fescue seeds. Table S8. Bacterial species significantly decreased in the rectum microbiome following toxic tall fescue supplementation. Figure S1. Differentially abundant microbial species in the rectum microbiome before and after fescue toxicosis treatment. Figure S2. Top 20 most abundant bacterial families and species in the cattle rectum microbiome before and after toxic tall fescue seed treatment. Data S1. Relative abundance of bacterial phyla in the cattle rectum microbiome before and after toxic tall fescue seed treatment. Data S2. Relative abundance of bacterial families in the cattle rectum microbiome before and after toxic tall fescue seed treatment. Data S3. Relative abundance of bacterial genera in the cattle rectum microbiome before and after toxic tall fescue seed treatment. Data S4. Relative abundance of bacterial species in the cattle rectum microbiome before and after toxic tall fescue seed treatment. Data S5. Annotated antimicrobial resistance genes identified in the cattle rectum microbiome.
