# Supplementary material for: Metagenomic Analysis Revealed Significant Changes in the Beef Cattle Rectum Microbiome Under Fescue Toxicosis
Source: Biology (Basel). 2025 Sep 5;14(9):1197. doi: 10.3390/biology14091197 (PMC12466995; doi:10.3390/biology14091197)

Figure S1. Differentially abundant microbial species in the rectum microbiome before and after fescue toxicosis treatment.

(**A-B**) Heatmaps of the relative frequency for the significantly increased (**A**) and decreased (**B**) species in cattle rectum microbiome after treatment. The species names are shown on the right (***blue***: top featured species after treatment; ***red***: top featured species before treatment).

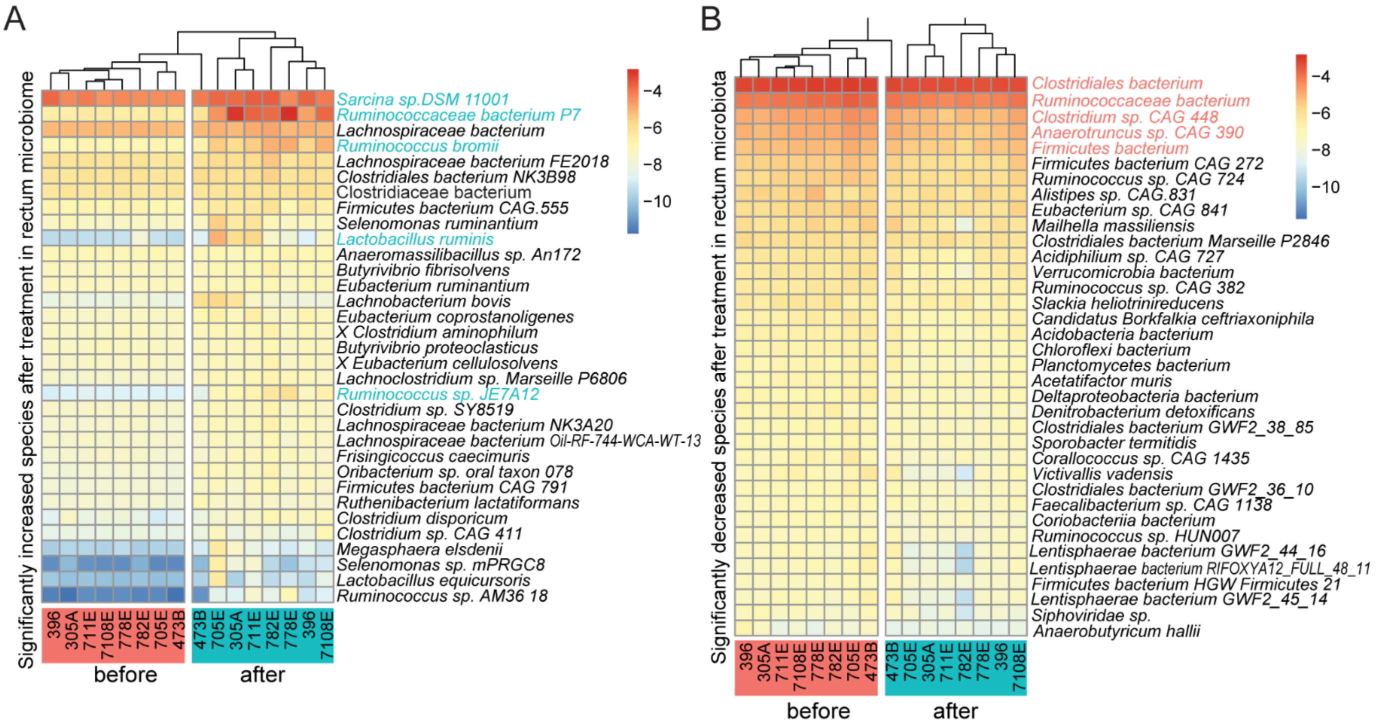

Figure S2. Top 20 most abundant bacterial families and species in the cattle rectum microbiome before and after toxic tall fescue seed treatment.

(**A-B**) Heatmap of relative frequency for the top 20 most abundant bacteria families (**A**) and species (**B**). The taxa names were rank-ordered with the most abundant taxon on the left.

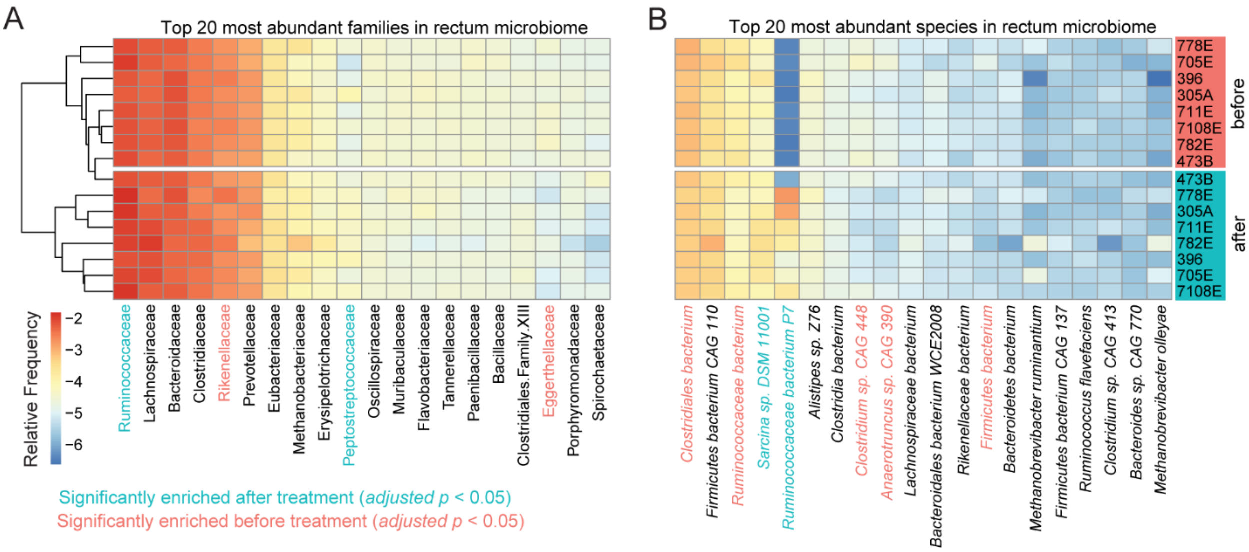

Supplement: Supplementary file 1 [file biology-14-01197-s001.zip › FigureS1-S2.pdf]
