# Supplementary material for: Metagenomic Analysis Revealed Significant Changes in the Beef Cattle Rectum Microbiome Under Fescue Toxicosis
Source: Biology (Basel). 2025 Sep 5;14(9):1197. doi: 10.3390/biology14091197 (PMC12466995; doi:10.3390/biology14091197)
Supplement: Supplementary file 1 [file biology-14-01197-s001.zip › TableS1-S8.pdf]

Table S1. Genotypic classification of tall fescue toxicosis tolerance using T-snip assay in cattle enrolled for rectal metagenomic profiling.

Animal ID	Category	Tolerance index	Genotype
7108 E	Heifer	*	Susceptible
711 E	Heifer	*	Susceptible
396	Cow	*	Susceptible
305 A	Cow	*	Susceptible
778 E	Heifer	****	Tolerant
782 E	Heifer	****	Tolerant
705 E	Heifer	****	Tolerant
473 B	Cow	****	Tolerant

Table S2. Bodyweight trajectories of individual cattle during the 4-week toxic fescue seed feeding experiment.

Animal ID	BW-week0	BW-week1	BW-week2	BW-week3	BW-week4
7108 E	452	484	429	447	456
711 E	526	547	487	514	534
396	547	562	528	532	542
305 A	712	703	672	685	692
778 E	444	466	425	443	450
782 E	485	504	487	499	519
705 E	533	547	505	517	535
473 B	671	673	658	665	676

Table S3. Whole-genome shotgun metagenomic sequencing yield and quality control metrics for cattle rectum/fecal samples.

Animal ID	total number of reads	total yield (Gbp)	% adapters	% host sequences	% viral sequences
305A_before	60,900,346	9.20	2.08%	0.49%	0.03%
305A_after	61,553,984	9.29	2.45%	1.79%	0.03%
396_before	28,935,274	4.37	3.06%	3.57%	0.02%
396_after	42,926,490	6.48	3.37%	2.18%	0.03%
473B_before	76,646,644	11.57	2.54%	20.61%	0.02%
473B_after	80,735,388	12.19	1.97%	1.11%	0.03%
705E_before	96,039,156	14.50	1.62%	18.15%	0.03%
705E_after	98,536,138	14.88	1.89%	1.52%	0.03%
7108E_before	76,035,880	11.48	2.14%	1.18%	0.03%
7108E_after	79,592,532	12.02	1.92%	0.72%	0.03%
711E_before	66,171,776	9.99	1.87%	3.38%	0.03%
711E_after	68,541,294	10.35	2.37%	11.51%	0.02%
778E_before	55,970,182	8.45	2.10%	18.10%	0.02%
778E_after	51,735,790	7.81	2.58%	2.31%	0.03%
782E_before	51,581,368	7.79	2.30%	5.29%	0.03%
782E_after	50,747,222	7.66	2.39%	2.84%	0.03%

Table S4. Read mapping statistics of rectum metagenomes against the assembled microbial reference database.

Animal ID	# of raw reads	# of filtered reads	# of uniquely mapped reads	mapping percentage
305A_before	60,900,346	50,716,714	44,639,229	88.02%
305A_after	61,553,984	49,013,890	45,221,899	92.26%
396_before	28,935,274	19,355,664	16,826,064	86.93%
396_after	42,926,490	30,929,976	27,673,398	89.47%
473B_before	76,646,644	41,005,674	36,203,066	88.29%
473B_after	80,735,388	55,408,398	50,026,954	90.29%
705E_before	96,039,156	45,086,146	43,171,426	95.75%
705E_after	98,536,138	66,020,296	62,610,669	94.84%
7108E_before	76,035,880	57,162,316	52,503,540	91.85%
7108E_after	79,592,532	55,032,980	52,110,816	94.69%
711E_before	66,171,776	49,244,496	44,988,422	91.36%
711E_after	68,541,294	43,360,052	39,830,080	91.86%
778E_before	55,970,182	32,834,628	29,880,100	91.00%
778E_after	51,735,790	40,359,064	37,721,050	93.46%
782E_before	51,581,368	36,948,830	33,826,790	91.55%
782E_after	50,747,222	38,852,638	36,854,255	94.86%

Table S5. Top 20 most abundant bacterial families in the cattle rectum microbiome before and after fescue toxicosis treatment.

rank	family name	before	after	before STDEV	after STDEV	NCBI ID
1	Ruminococcaceae	12.90%	14.77%	1.10%	1.62%	216572
2	Lachnospiraceae	10.96%	12.36%	0.46%	1.62%	186803
3	Bacteroidaceae	11.92%	11.17%	0.75%	0.82%	815
4	Clostridiaceae	8.78%	9.37%	0.31%	0.91%	31979
5	Rikenellaceae	7.52%	7.81%	0.62%	0.92%	171550
6	Prevotellaceae	6.29%	6.17%	0.54%	0.89%	171552
7	Eubacteriaceae	2.70%	2.89%	0.08%	0.30%	186806
8	Methanobacteriaceae	2.11%	2.36%	0.55%	0.88%	2159
9	Erysipelotrichaceae	1.42%	1.64%	0.08%	0.36%	128827
10	Peptostreptococcaceae	1.06%	1.38%	0.42%	0.25%	186804
11	Oscillospiraceae	1.24%	1.17%	0.06%	0.08%	216572
12	Muribaculaceae	1.22%	1.12%	0.07%	0.14%	2005473
13	Flavobacteriaceae	1.23%	1.10%	0.11%	0.22%	49546
14	Tannerellaceae	1.19%	1.09%	0.08%	0.13%	2005525
15	Paenibacillaceae	1.25%	1.02%	0.08%	0.14%	186822
16	Bacillaceae	1.13%	1.00%	0.06%	0.08%	186817
17	Clostridiales Family XIII. Incertae Sedis	1.03%	1.06%	0.08%	0.11%	543314
18	Eggerthellaceae	1.14%	0.78%	0.17%	0.12%	1643826
19	Porphyromonadaceae	0.99%	0.88%	0.08%	0.14%	171551
20	Spirochaetaceae	0.93%	0.75%	0.11%	0.29%	137

Table S6. Top 20 most abundant bacterial species identified in the cattle rectum microbiome across treatment conditions.

rank	species name	before	after	before STDEV	after STDEV	NCBI ID
1	*Clostridiales bacterium,* unclassified	4.78%	4.07%	0.28%	0.18%	1898207
2	*Firmicutes bacterium CAG:110*	3.26%	3.70%	0.43%	0.73%	1263000
3	*Ruminococcaceae bacterium,* unclassified	2.34%	1.79%	0.42%	0.31%	2485925
4	*Sarcina sp. DSM 11001*	1.62%	2.42%	0.41%	0.60%	1798184
5	*Ruminococcaceae bacterium P7*	0.17%	2.87%	0.01%	2.27%	1200751
6	*Alistipes sp. Z76*	1.15%	1.22%	0.15%	0.14%	2304565
7	*Clostridia bacterium,* unclassified	0.92%	0.85%	0.03%	0.06%	2044939
8	*Clostridium sp. CAG:448*	0.99%	0.69%	0.20%	0.15%	1262808
9	*Anaerotruncus sp. CAG:390*	0.83%	0.60%	0.13%	0.12%	1262703
10	*Lachnospiraceae bacterium,* unclassified	0.64%	0.73%	0.04%	0.08%	1898203
11	*Bacteroidales bacterium WCE2008*	0.71%	0.65%	0.11%	0.09%	1945891
12	*Rikenellaceae bacterium,* unclassified	0.54%	0.60%	0.08%	0.11%	2049048
13	*Firmicutes bacterium,* unclassified	0.62%	0.49%	0.08%	0.08%	1879010
14	*Bacteroidetes bacterium,* unclassified	0.58%	0.51%	0.08%	0.13%	1898104
15	*Methanobrevibacter ruminantium*	0.36%	0.55%	0.16%	0.29%	83816
16	*Firmicutes bacterium CAG:137*	0.42%	0.46%	0.05%	0.10%	1263004
17	*Ruminococcus flavefaciens*	0.44%	0.45%	0.04%	0.06%	1265
18	*Clostridium sp. CAG:413*	0.42%	0.41%	0.06%	0.12%	1262803
19	*Bacteroides sp. CAG:770*	0.41%	0.39%	0.06%	0.06%	1262751
20	*Methanobrevibacter olleyae*	0.32%	0.48%	0.13%	0.25%	294671

Table S7. Bacterial species significantly enriched in the rectum microbiome after exposure to endophyte-infected tall fescue feed.

rank	species name	before	after	before STDEV	after STDEV	NCBI ID
1	*Ruminococcaceae bacterium P7*	0.17%	2.87%	0.01%	2.27%	1200751
2	*Sarcina sp. DSM 11001*	1.62%	2.42%	0.41%	0.60%	1798184
3	*Lachnospiraceae bacterium,* unclassified	0.64%	0.73%	0.04%	0.08%	1898203
4	*Ruminococcus bromii*	0.11%	0.51%	0.01%	0.24%	40518
5	*Lachnospiraceae bacterium FE2018*	0.24%	0.33%	0.02%	0.09%	1410624
6	*Clostridiales bacterium NK3B98*	0.24%	0.30%	0.01%	0.06%	877414
7	*Clostridiaceae bacterium,* unclassified	0.21%	0.24%	0.02%	0.02%	1898204
8	*Firmicutes bacterium CAG:555*	0.12%	0.18%	0.00%	0.04%	1263030
9	*Selenomonas ruminantium*	0.07%	0.16%	0.01%	0.13%	971
10	*Lactobacillus ruminis*	0.01%	0.21%	0.01%	0.27%	1623
11	*Anaeromassilibacillus sp. An172*	0.10%	0.11%	0.01%	0.01%	1965570
12	*Butyrivibrio fibrisolvens*	0.08%	0.10%	0.00%	0.02%	831
13	*Eubacterium ruminantium*	0.08%	0.10%	0.00%	0.01%	42322
14	*Lachnobacterium bovis*	0.04%	0.14%	0.01%	0.14%	140626
15	*Eubacterium coprostanoligenes*	0.07%	0.11%	0.00%	0.04%	290054
16	*[Clostridium] aminophilum*	0.07%	0.10%	0.00%	0.02%	1526
17	*Butyrivibrio proteoclasticus*	0.07%	0.08%	0.01%	0.01%	43305
18	*[Eubacterium] cellulosolvens*	0.07%	0.08%	0.00%	0.02%	29322
19	*Lachnoclostridium sp. Marseille-P6806*	0.05%	0.09%	0.00%	0.02%	2364793
20	*Ruminococcus sp. JE7A12*	0.02%	0.13%	0.00%	0.08%	2564099
21	*Clostridium sp. SY8519*	0.06%	0.08%	0.00%	0.02%	1042156
22	*Lachnospiraceae bacterium NK3A20*	0.05%	0.09%	0.00%	0.02%	877406
23	*Lachnospiraceae bacterium Oil+RF-744-WCA-WT-13*	0.06%	0.07%	0.00%	0.02%	2606636
24	*Frisingicoccus caecimuris*	0.05%	0.07%	0.01%	0.02%	1796636
25	*Oribacterium sp. oral taxon 078*	0.05%	0.08%	0.00%	0.02%	652706
26	*Firmicutes bacterium CAG:791*	0.04%	0.08%	0.00%	0.02%	1262993
27	*Ruthenibacterium lactatiformans*	0.05%	0.06%	0.00%	0.02%	1550024
28	*Clostridium disporicum*	0.03%	0.07%	0.01%	0.02%	84024
29	*Clostridium sp. CAG:411*	0.03%	0.05%	0.00%	0.03%	1262802
30	*Megasphaera elsdenii*	0.01%	0.04%	0.00%	0.06%	907
31	*Selenomonas sp. mPRGC8*	0.00%	0.04%	0.00%	0.05%	2606905
32	*Lactobacillus equicursoris*	0.00%	0.03%	0.00%	0.06%	420645
33	*Ruminococcus sp. AM36-18*	0.00%	0.03%	0.00%	0.03%	2293209

Table S8. Bacterial species significantly decreased in the rectum microbiome following toxic tall fescue supplementation.

rank	species name	before	after	before STDEV	after STDEV	NCBI ID
1	*Clostridiales bacterium,* unclassified	4.78%	4.07%	0.28%	0.18%	1898207
2	*Ruminococcaceae bacterium,* unclassified	2.34%	1.79%	0.42%	0.31%	2485925
3	*Clostridium sp. CAG:448*	0.99%	0.69%	0.20%	0.15%	1262808
4	*Anaerotruncus sp. CAG:390*	0.83%	0.60%	0.13%	0.12%	1262703
5	*Firmicutes bacterium,* unclassified	0.62%	0.49%	0.08%	0.08%	1879010
6	*Firmicutes bacterium CAG:272*	0.46%	0.35%	0.07%	0.09%	1263015
7	*Ruminococcus sp. CAG:724*	0.42%	0.31%	0.07%	0.05%	1262966
8	*Alistipes sp. CAG:831*	0.39%	0.29%	0.11%	0.06%	1262698
9	*Eubacterium sp. CAG:841*	0.37%	0.29%	0.06%	0.06%	1262894
10	*Mailhella massiliensis*	0.33%	0.22%	0.07%	0.11%	1903261
11	*Clostridiales bacterium Marseille-P2846*	0.30%	0.25%	0.03%	0.04%	1852363
12	*Acidiphilium sp. CAG:727*	0.22%	0.16%	0.02%	0.03%	1262689
13	*Verrucomicrobia bacterium,* unclassified	0.21%	0.15%	0.04%	0.06%	2026799
14	*Ruminococcus sp. CAG:382*	0.20%	0.15%	0.02%	0.02%	1262957
15	*Slackia heliotrinireducens*	0.20%	0.14%	0.03%	0.03%	84110
16	*Candidatus Borkfalkia ceftriaxoniphila*	0.16%	0.13%	0.02%	0.02%	2508949
17	*Acidobacteria bacterium,* unclassified	0.16%	0.12%	0.01%	0.02%	1978231
18	*Chloroflexi bacterium,* unclassified	0.14%	0.11%	0.01%	0.02%	2026724
19	*Planctomycetes bacterium,* unclassified	0.13%	0.10%	0.02%	0.03%	2026780
20	*Acetatifactor muris*	0.12%	0.10%	0.01%	0.01%	879566
21	*Deltaproteobacteria bacterium,* unclassified	0.13%	0.10%	0.02%	0.02%	2026735
22	*Denitrobacterium detoxificans*	0.13%	0.09%	0.02%	0.02%	79604
23	*Clostridiales bacterium GWF2_38_85*	0.12%	0.09%	0.01%	0.02%	1797683
24	*Sporobacter termitidis*	0.11%	0.09%	0.01%	0.01%	44749
25	*Corallococcus sp. CAG:1435*	0.12%	0.08%	0.03%	0.01%	1262867
26	*Victivallis vadensis*	0.12%	0.07%	0.04%	0.05%	172901
27	*Clostridiales bacterium GWF2_36_10*	0.11%	0.08%	0.01%	0.02%	1797682
28	*Faecalibacterium sp. CAG:1138*	0.10%	0.08%	0.01%	0.01%	1262896
29	*Coriobacteriia bacterium,* unclassified	0.09%	0.07%	0.01%	0.01%	2052159
30	*Ruminococcus sp. HUN007*	0.10%	0.06%	0.04%	0.01%	1514668
31	*bacterium*	0.09%	0.07%	0.01%	0.01%	1869227
32	*Lentisphaerae bacterium GWF2_44_16*	0.10%	0.06%	0.03%	0.04%	1798571
33	*Lentisphaerae bacterium,* unclassified *RIFOXYA12_FULL_48_11*	0.09%	0.06%	0.03%	0.03%	1798578
34	*Firmicutes bacterium HGW-Firmicutes-21*	0.09%	0.06%	0.01%	0.01%	2013783
35	*Lentisphaerae bacterium GWF2_45_14*	0.09%	0.06%	0.02%	0.03%	1798572
36	*Siphoviridae sp.*	0.08%	0.04%	0.02%	0.02%	2170413
37	*Anaerobutyricum hallii*	0.06%	0.03%	0.03%	0.00%	39488
